# Motivations of Sports Volunteers at Mass Endurance Events: A Case Study of Poznan

**DOI:** 10.3390/sports13080255

**Published:** 2025-08-01

**Authors:** Milena Michalska, Mateusz Grajek, Mateusz Rozmiarek

**Affiliations:** 1 Department of Sports Tourism, Faculty of Physical Culture Sciences, Poznan University of Physical Education, 61-871 Poznan, Poland; 2 Department of Public Health, Faculty of Public Health in Bytom, Medical University of Silesia in Katowice, 41-902 Bytom, Poland; mgrajek@sum.edu.pl

**Keywords:** volunteering, motivation, sport, running, endurance events, volunteer management, sports event management, sports volunteering experience, Poznan, Poland

## Abstract

Sport volunteering plays an important role in achieving the goals of sustainable development by supporting the social dimension of sustainability, fostering social integration, and promoting a healthy lifestyle. However, there is a lack of systematic research in Poland on the motivations of sport volunteers, particularly in the context of mass endurance events. This study employed a quantitative, cross-sectional design involving 148 sport volunteers engaged in mass endurance events in Poznan, Poland. To measure motivation, the Polish adaptation of the VMS-ISE scale was used. Data analysis was conducted using one-way analysis of variance (ANOVA). The results showed that volunteer motivations were relatively homogeneous regardless of gender and education level, with the exception of passion for sport, which was significantly stronger among men (*p* = 0.037). Significant differences were found based on place of residence: residents of medium-sized cities demonstrated the highest motivation for personal development (*p* < 0.001), whereas individuals from rural areas exhibited stronger patriotism, a greater need for interpersonal interaction, and a higher valuation of external rewards (*p* < 0.05). The motivations of sport volunteers in Poland are complex and sensitive to environmental factors. Understanding these differences allows for better alignment of recruitment and volunteer management strategies, which can enhance both the effectiveness and sustainability of volunteer engagement. It is recommended to develop volunteer programs that take into account the demographic and socio-cultural characteristics of participants.

## 1. Introduction

Sport volunteering is increasingly perceived not only as a component of organizational support for events but also as a significant element in achieving the goals of sustainable development [1]. The engagement of volunteers in sporting events contributes to building social cohesion, fostering civic engagement, and strengthening social capital, aligning with the social dimension of sustainability [2,3]. Participation in volunteering fosters the development of interpersonal relationships, strengthens mutual trust, and enhances the sense of belonging to the local community. Such engagement creates a space for grassroots social integration and the formation of lasting bonds based on cooperation and shared goals [4]. On the other hand, proper volunteer management can also support environmental goals—for example, through ecological education of volunteers, promoting low-emission transport among event participants, or involvement in activities aimed at reducing waste during mass events [5]. In the long term, sport volunteering—particularly when rooted in community values—can contribute to a more responsible and conscious organization of sporting events that minimize negative environmental impacts while reinforcing local social bonds. Thus, sport volunteering becomes not only a logistical resource but also a practical tool for implementing the principles of sustainable development.

Volunteers play a key role in the organization and atmosphere of sporting events, influencing participants on multiple levels. Among other things, they can have emotional and motivational effects, particularly on individuals experiencing the event for the first time, whether as participants or spectators. Positive experiences gained during the event often encourage volunteers to re-engage, highlighting their contribution to participant motivation and overall event satisfaction. Moreover, the way organizers manage volunteers—including the provision of feedback, support, and training—significantly affects the satisfaction of both the volunteers themselves and the event participants [6].

Sport volunteering stands out from other forms of volunteering, among other reasons, due to the emotions experienced by participants during sporting events [7]. Volunteers are often interested in a given sport discipline and enthusiastically support the athletes, helping to create a positive and spirited atmosphere at events. Furthermore, through their actions, they promote values such as fair play, respect for others, and appreciation for different cultures, which enhances the socio-cultural significance of sporting events [7,8]. The aspect of educational activity is also important. Volunteers often act as educators, providing participants and the public with knowledge about safety and healthy lifestyles. Volunteering creates a space for practical acquisition of skills: communication, management, working with people or organization. Sports events are often intense experiences that pose challenges—thanks to them, volunteers learn to act under pressure, make decisions and adapt to situations, which supports the development of leadership and motivational competences [9].

A dimension of pure curiosity can be understood as cognitive motivation, or the desire to explore a new environment, learn, and participate in unique events. In studies on volunteering at major international sporting events, this motivation has been linked to the willingness to learn, participate in something unique, and observe the event “from the inside,” even without prior ties to sport or event organizers. Exploratory motivation includes the desire to learn, get new experiences and see what it’s like to organize an event from scratch. Cognitive interest and the desire to learn new experiences as one of the key internal motivations for volunteering [10].

Volunteers form the foundation of many sporting events, and their commitment—often altruistic and grounded in a strong sense of community—can be effectively strengthened when organizers recognize and respond to their needs and expectations [11,12]. Research into volunteer motivations supports not only recruitment and retention efforts but also the design of more sustainable human capital management strategies for sporting events [13,14]. Understanding whether the primary driving force is the desire to gain experience, social belonging, altruism, or the will to support specific initiatives allows for the creation of volunteer programs that are more effective and long-lasting [15,16,17,18]. Thus, the study of volunteer motivations is not merely an organizational tool but also a key component in fostering conscious, responsible, and sustained civic engagement in sport. Volunteers often state that they are motivated by a desire to represent their city or region. Participating in a regional, national or international sporting event can generate a strong sense of pride in local belonging and a desire to promote one’s place of residence [19].

Sport volunteering plays a particularly important role during endurance-based mass running events [20,21,22], as such events often involve hundreds or even thousands of participants and spectators, requiring efficient organization and support on multiple levels. Event volunteers are responsible for a wide range of tasks—from managing refreshment stations, securing the route, and assisting with registration to providing logistical support [23,24]. Their involvement is essential for ensuring the safety, comfort, and positive experience of participants [25]. Due to their popularity and social nature, endurance events also offer a unique opportunity to implement sustainable development principles by promoting healthy lifestyles, fostering community integration, and building social capital.

At the international level, research on the motivations of sport volunteers is already a well-established field, encompassing both mass events and smaller local initiatives. Numerous studies conducted across different parts of the world have deepened our understanding of sport volunteers’ motivations in diverse cultural and social contexts, taking into account regional differences and event types [26,27,28,29,30,31]. At the same time, diagnostic tools have been developed to identify key sources of motivation, analyze their variability over time, and assess the influence of various factors on the duration and quality of volunteer engagement [32,33,34,35,36].

In Poland, however, this area of research—despite the growing popularity of sport volunteering—remains at a relatively early stage of academic development. In recent years, the first publications by Polish scholars, mainly Bańbuła [37,38] and Rozmiarek [39,40,41,42], have appeared on the international academic market. Nonetheless, there is still a shortage of comprehensive quantitative and qualitative studies that would examine volunteer motivations in the context of specific sporting events, cultural conditions, or management strategies. As a result, both event organizers and institutions supporting sport volunteering have limited knowledge of what drives individuals to participate and how to effectively build long-term engagement. This highlights an urgent need to intensify research in this area—not only to bridge the gap with international scholarship but also to generate knowledge tailored to the specific characteristics of the Polish sport and social environment. Influence of local authorities and public institutions is not only to organize the event, but also to mobilize the community to participate. City programs, promotional campaigns or offering volunteering certificates have a real impact on the decision to engage in this type of activity [43].

In line with current research trends in sports volunteering [44], the aim of this study was to examine the motivations of individuals engaged in sports volunteering, with a particular focus on volunteers working at mass endurance events organized in Poznan, Poland. This analysis allowed for a better understanding of the factors influencing the decision to participate and the nature of engagement, which constitutes an important step toward effective management and development of sports volunteering in the Polish context.

## 2. Materials and Methods

### 2.1. Study Design and Participants

The present study is a descriptive, quantitative, cross-sectional study that sampled 148 volunteers (81 women and 67 men; 58.8% with higher education, 36.5% with secondary, and 4.7% with primary or vocational education; mean BMI = 22.9, SD = 2.8, with no participants classified as obese) from Poznan, Poland. The study was conducted between June 2024 and April 2025. The survey questionnaire was completed by sports volunteers during events such as: Enea Junior Poznan Triathlon, PKO Poznan Half Marathon, Enea Ironman Poznan and Wings For Life World Run in Poznan. These events, due to their varying scale and level of difficulty, attract anywhere from several hundred participants (triathlon) to even a few thousand (Wings for Life, Ironman) or several tens of thousands (half marathon). The data were collected during or immediately after each of the listed events, depending on organizational logistics. Since the events were held on different dates, data collection was distributed across multiple time points.

The study was conducted by one of the authors of this article. Participants were recruited on-site at volunteer coordination points and via internal social media groups used for event-related communication. All participants completed the questionnaire individually, in similar conditions. Participants were informed about the purpose of the study, the voluntary nature of their participation, and the anonymity of their responses. Standardized instructions were given to ensure consistency across events. Inclusion criteria for participation in the study included: (1) being at least 18 years old, (2) having an active volunteer role during one of the studied sporting events, and (3) providing informed consent to participate in the survey. Exclusion criteria were: (1) refusal to give informed consent, (2) incomplete responses to key parts of the questionnaire, and (3) participation in the event only as a spectator or staff member without a formal volunteer role. Informed verbal consent was obtained from all participants, as the study involved minimal risk, ensured full anonymity, and data were collected via self-administered questionnaires.

### 2.2. Research Instrument

The study, conducted using the diagnostic survey method and the questionnaire technique, employed a survey questionnaire divided into two parts. In the first part, participants were asked about their gender (male, female), education, place of residence, age, weight (kg), and height (cm), with weight and height used to calculate Body Mass Index (BMI). The second part of the questionnaire focused on factors motivating volunteers. Respondents were presented with a set of 37 closed-ended statements regarding potential motivations for engaging in sports volunteering. They evaluated these statements using a 7-point Likert scale, where 1 indicated “not a reason at all” and 7 signified a “very important reason”.

Volunteer motivations were measured using the Polish adaptation of the VMS-ISE (Volunteer Motivation Scale for International Sporting Events). This is a specialized tool developed to assess the motivations of volunteers participating in international sporting events. The scale was originally created by Bang and Chelladurai [45], and the Polish adaptation and validation (referred to as VMS-ISE.PL, published in *BMC Sports Science, Medicine and Rehabilitation*) were conducted by Rozmiarek and colleagues [46]. The original version of the scale consisted of six motivational factors: *Expression of Values, Patriotism, Interpersonal Contacts, Career Orientation, Personal Growth, Extrinsic Rewards*. In later years, an additional seventh factor—*Love of Sport*—was added [47].

### 2.3. Data Analysis

The analysis of the results was carried out using Microsoft Excel, while the statistical interpretation was performed using Statistica 13.3 (StatSoft, Kraków, Poland). Prior to performing any inferential analyses, the assumptions of normality and homogeneity of variances were assessed. Shapiro–Wilk tests confirmed that most of the motivational and dietary-related variables were approximately normally distributed (*p* > 0.05). Levene’s tests were used to assess the homogeneity of variances. For comparisons between two independent groups (e.g., gender), independent-samples *t*-tests were used. For comparisons involving more than two groups (e.g., education level or place of residence), one-way ANOVA was applied depending on the result of the Levene’s test. Mean values and standard deviations were calculated for all relevant variables. In cases where normality or variance assumptions were violated but robust tests were not available, results were interpreted with caution. Pearson’s correlation coefficients were calculated to assess relationships between motivational and nutritional variables, assuming approximate normality. The level of statistical significance was set at α = 0.05. In the tables included in the article, an asterisk (*) indicates statistical significance at the *p* < 0.05 level, while a double asterisk (**) indicates statistical significance at the *p* < 0.01 level.

## 3. Results

The statistical analysis conducted on the dataset collected from sports volunteers revealed a number of relationships between sociodemographic variables and the identified motivational constructs. In the present study, the predictor variables included: gender, age, place of residence, level of education, and body mass index (BMI), calculated based on respondents’ height and weight.

Table 1 presents the effect of gender on the motivational structure. Most motivational categories did not show statistically significant differences between women and men, suggesting a relatively homogeneous motivational profile regardless of gender. An exception was the motivation rooted in the *Love of Sport*, which was significantly higher among men (M = 5.83, SD = 1.05) compared to women (M = 5.38, SD = 1.46), with a *p*-value of 0.037. This result implies that men, statistically speaking, are more often driven by an intrinsic passion for sport as a motive for engaging in volunteer activities.

Table 2 presents the results of sports volunteers’ motivation in relation to their level of education. No statistically significant differences were found in any of the motivational categories analyzed with respect to educational attainment. This suggests that the motivational structure of individuals engaging in sports volunteering remains relatively consistent regardless of their level of education. It can therefore be assumed that other factors—such as personal interests, individual values, or prior volunteering experience—play a more substantial role than formal education level.

Table 3 presents the results of sports volunteers’ motivation depending on their place of residence. The classification into villages, small towns (up to 20,000 inhabitants), medium-sized towns (20,000–100,000 inhabitants), and large cities (over 100,000 inhabitants) is commonly used in Poland and is applied both in publications of the Central Statistical Office and in academic studies concerning settlement structure and socio-demographic analyses [48]. When analyzing the impact of place of residence, notably deeper differences emerged. Respondents from medium-sized cities (20,000–100,000 inhabitants) achieved the highest scores in the area of developmental motivation (*Personal Growth*)—the mean in this group was 6.09 (SD = 0.76), compared to residents of large cities who scored 5.38 (SD = 1.01), residents of small towns (M = 5.11, SD = 1.56), and rural inhabitants (M = 4.80, SD = 1.23). These differences were statistically significant, which may indicate a greater importance of self-realization and personal development aspects for individuals from areas with limited access to professional training, events, and educational initiatives. In terms of patriotic motivation (*Patriotism*), respondents living in rural areas achieved the highest mean score (M = 4.71, SD = 1.74), followed by residents of medium-sized cities (M = 4.25, SD = 1.82), while inhabitants of large cities scored lower on this scale (M = 3.40, SD = 1.49). The significance of these differences was confirmed by ANOVA analysis. Such a distribution may indicate a stronger attachment to national identity and community values among people living in smaller centers. Post-hoc comparisons using the Tukey HSD test indicated that participants from rural areas differed significantly in terms of *Patriotism* and *Extrinsic Rewards* compared to those from large cities (*p* < 0.05). Similarly, the highest *Personal Growth* motivation was found among residents of medium-sized towns, significantly higher than in small towns and rural areas (*p* < 0.01).

Differences were also observed in the area of interpersonal motivation (*Interpersonal Contacts*) depending on place of residence. Respondents from medium-sized cities and rural areas showed a higher need for contact with others (M = 6.21, SD = 0.69 and M = 6.11, SD = 0.89, respectively), which can be interpreted as an expression of the desire for social integration and strengthening relationships, whose deficit might be more strongly felt in less urbanized areas. Interestingly, a similar trend—though of a slightly different nature—was also found in motivation related to extrinsic rewards (*Extrinsic Rewards*). The highest mean score was recorded by rural residents (M = 4.29, SD = 2.21), which may suggest a greater significance of material gratifications (such as gadgets, tickets, meals) as factors reinforcing the decision to engage in volunteering. This result may indicate the existence of socio-economic barriers that make extrinsic rewards a more important aspect for volunteers from less privileged areas.

Table 4 visualizes the correlations between sports volunteers’ motivations and age as well as body mass index (BMI). The analysis of the impact of age, conducted using Pearson’s correlation, showed that among all motivational categories, only *Patriotism* demonstrated a significant positive correlation with age (r = 0.280, *p* = 0.001). This relationship may indicate an increasing sense of civic responsibility and a stronger attachment to national values and the need to represent the country through participation in sporting events as people grow older. In other cases, no significant associations were observed, suggesting the relative independence of most motivational dispositions from the biological age of participants.

Equally interesting were the results of the BMI analysis. Among the seven motivational categories, only *Love of Sport* showed a significant negative correlation with this indicator (r = −0.205, *p* = 0.012). This result suggests that individuals with higher BMI are less driven by a passion for sport as a reason for engaging in volunteering. This may be due both to a lower level of physical activity among these individuals and a lesser identification with a sporty lifestyle, which in turn influences the choice of more external or instrumental motivations.

In addition to statistical significance, effect sizes were calculated to assess the practical importance of findings. For ANOVA, eta-squared (η^2^) was reported. For significant results, effect sizes ranged from small (η^2^ = 0.03) to moderate (η^2^ = 0.07). For correlations, the squared Pearson coefficient (r^2^) indicated that age explained approximately 7.8% of the variance in *Patriotism*, and BMI accounted for 4.2% of the variance in *Love of Sport*.

## 4. Discussion

The analysis of sports volunteers’ motivations in the context of sociodemographic variables revealed a complex picture of factors influencing their engagement [28,29,33,36], yet it is extremely important for the effective management of events [35,49,50]. According to the results, volunteers’ motivations show relative stability regarding gender and education level, while more significant differences appear in the context of place of residence and age.

The examined motivational categories did not differ significantly between women and men, suggesting that the motivational mechanisms driving involvement in sports volunteering are largely gender-universal, although an exception was the *Love of Sport*, which was stronger among men. This finding is fully consistent with previous studies conducted by VanSickle et al. [51], as well as the results of Ye and colleagues [52]. It may reflect cultural or social differences related to the perception of sport, where men more often identify with the sporty lifestyle and derive greater intrinsic satisfaction from it. Interestingly, Vetitnev et al. [27] reported quite different results in this regard, showing that female volunteers express a greater desire for *Patriotism*, *Interpersonal Contacts*, and *Career Orientation* than male volunteers. The latter motivation among women was also noted by Jarvis and Blank [53]. In turn, the study conducted by Bomber [54], carried out at a large-scale non-sporting event with a predominantly female group of respondents, showed that their motivation to volunteer may also stem from spiritual values, a sense of unity, and a desire for personal development. This is important; however, motivating people to engage in volunteering cannot rely solely on moral values, as indicated by Piechota [55]. The obtained results, compared with other studies, thus support Koutrou’s thesis [56] that volunteers at large events are motivated by a variety of factors, other than those motivating volunteers who, for example, work at local sports events or sports clubs, and that the influence of these factors can vary significantly from person to person. Due to these differences, further research into the influence of gender on motivations in the context of local environments seems warranted.

The results regarding the influence of education level on motivations are consistent with the findings of Vetitnev et al. [27], as in both cases no statistically significant differences were found. This may suggest that engagement in volunteering is often determined more by personal values and life experiences than by formal education.

The most interesting results concern the impact of place of residence. Respondents from smaller towns and rural areas showed higher levels of patriotic, interpersonal, and extrinsic rewards-related motivations, which may result from the greater role of local community and social bonds in these areas [57,58]. In particular, the stronger attachment to patriotic values among rural residents may reflect a more pronounced sense of local and national identity [59]. The high level of interpersonal motivation and the importance of material gratifications in this group suggest that volunteering plays a significant integrative and compensatory role, helping to mitigate social and economic deficits characteristic of less urbanized areas.

These findings may also be rooted in broader cultural factors. In many rural communities, social life is more tightly woven around local traditions, institutions, and values such as patriotism or mutual aid [60,61,62]. The sense of belonging and identity is often more place-bound, which can translate into a stronger desire to support national or local events perceived as meaningful to the community. Moreover, in areas with limited access to job opportunities or formal education, extrinsic incentives such as event-branded merchandise or official certificates may be perceived not only as a reward but also as symbolic recognition of one’s role and contribution. Such motivations can reflect a culturally embedded understanding of volunteering as both a civic duty and a way to gain social visibility and respect.

The correlation analysis showed that age positively correlates only with patriotic motivation, which may indicate an increasing civic awareness and sense of responsibility for the community as people age. The lack of other significant age-related associations suggests that most motivations remain relatively stable across the age spectrum of participants. Meanwhile, the negative correlation between BMI and the motivation *Love of Sport* suggests that individuals with higher body weight are less driven by passion for sport, which may be related to lower physical activity or lesser identification with a sporty lifestyle [63,64].

## 5. Study Limitations and Practical Implications

Although the conducted study provided valuable insights into the motivations of sports volunteers, several important limitations should be considered, which may affect the generalizability and interpretation of the results. First, the sample consisted of 148 volunteers from a single city—Poznan—which limits the representativeness of the findings for the broader population of sports volunteers in Poland or other cultural contexts. This localized sample reduces the external validity of the study, and caution should be exercised when attempting to generalize the results beyond this specific geographic area. Second, the recruitment of participants was based on a non-probability, voluntary sampling method, which may introduce self-selection bias. This means that individuals who were more motivated, engaged, or had particular interest in volunteering might have been more likely to participate in the survey, potentially skewing the results. Such bias limits the ability to draw conclusions about the motivations of less engaged or less motivated volunteers. Third, the study’s cross-sectional design captures only a single point in time and does not allow for assessment of changes in volunteer motivations over time or the establishment of causal relationships. This limits the understanding of how motivations might evolve through different stages of volunteering or in response to specific events or contexts. Fourth, the study did not account for potential differences in volunteer motivations related to the characteristics of individual sporting events, such as event size, prestige, or type (e.g., triathlon vs. running events). These factors could influence volunteer engagement and motivational priorities but were not specifically analyzed in this study. Fifth, while basic sociodemographic variables were included, other potentially influential factors such as employment status, income level, previous volunteering experience, or cultural background were not assessed. These variables could provide a more nuanced understanding of what drives sports volunteering but remain unexplored in the current research. In light of these limitations, future research should aim to utilize multicenter or stratified sampling approaches, encompassing diverse geographic regions and event types to enhance representativeness and generalizability. Moreover, longitudinal study designs are recommended to track motivational dynamics over time and better understand causal mechanisms influencing volunteer engagement.

The study results provide important insights for sports event organizers and institutions responsible for recruiting and managing volunteers. The identified relative universality of motivations across gender suggests that initiatives promoting sports volunteering can be general in nature; however, it is worth considering the stronger significance of passion for sport among men, which could be leveraged in recruitment campaigns targeted at this group. On the other hand, the lack of significant motivational differences based on education level indicates that volunteer opportunities can be effectively offered to a wide audience without the need to tailor messages according to this factor. The obtained results can also serve as a foundation for designing more personalized volunteer support and reward programs that address dominant motives such as the desire to help, the need to establish social contacts, or personal development.

## 6. Conclusions

The study of motivations among sports volunteers involved in mass endurance events in Poland reveals that their motivations are diverse and largely influenced by their environment. Despite relative homogeneity in terms of gender and education, significant differences were observed based on place of residence—rural residents demonstrated stronger patriotic ties, a greater need for interpersonal contacts, and placed more value on external rewards, while residents of medium-sized cities were more oriented toward personal growth. These findings highlight the necessity to tailor recruitment and volunteer management strategies in sports to the demographic and cultural specifics of participants, which can contribute to increased engagement and sustainability of volunteer efforts. It is recommended to develop volunteer programs that take these differences into account to more effectively support sustainable development goals as well as promote social integration and a healthy lifestyle. Practically, these insights can inform policymakers and event organizers in designing targeted volunteer recruitment campaigns and strategies that address the unique motivational profiles of different communities, thereby enhancing volunteer satisfaction, performance, and long-term commitment.

## Figures and Tables

**Table 1:** Motivational results of sports volunteers from Poznan by gender.

Study Variable	Women (Mean ± SD)	Men (Mean ± SD)	t	*p*	Cohen’s d
Expression of Values	6.31 ± 0.88	6.28 ± 0.90	0.20	0.848	0.03
Patriotism	3.70 ± 1.69	3.70 ± 1.55	0.00	0.999	0.00
Interpersonal Contacts	5.72 ± 1.02	5.85 ± 0.91	−0.82	0.411	−0.13
Career Orientation	5.26 ± 1.36	5.42 ± 1.04	−0.85	0.419	−0.13
Personal Growth	5.65 ± 1.00	5.38 ± 1.10	1.55	0.119	0.25
Extrinsic Rewards	2.91 ± 1.56	3.26 ± 1.49	−1.39	0.158	−0.23
Love of Sport	5.38 ± 1.46	5.83 ± 1.05	−2.18	0.037 *	−0.35

**Table 2:** Motivational results of sports volunteers from Poznan by level of education.

Study Variables	Education	Mean ± SD	N	F	*p*
Expression of Values	Primary	6.06 ± 0.83	4	0.382	0.766
	Higher	6.36 ± 0.86	87		
	Vocational	6.42 ± 0.80	3		
	Secondary	6.22 ± 0.95	54		
Patriotism	Primary	4.60 ± 1.43	4	2.659	0.051
	Higher	3.94 ± 1.66	87		
	Vocational	4.00 ± 0.53	3		
	Secondary	3.23 ± 1.52	54		
Interpersonal Contacts	Primary	5.55 ± 1.28	4	0.327	0.806
	Higher	5.77 ± 0.94	87		
	Vocational	6.27 ± 0.64	3		
	Secondary	5.79 ± 1.03	54		
Career Orientation	Primary	6.12 ± 0.50	4	0.719	0.542
	Higher	5.28 ± 1.32	87		
	Vocational	5.00 ± 1.74	3		
	Secondary	5.38 ± 1.06	54		
Personal Growth	Primary	6.00 ± 0.62	4	0.383	0.766
	Higher	5.48 ± 1.06	87		
	Vocational	5.33 ± 1.72	3		
	Secondary	5.57 ± 1.04	54		
Extrinsic Rewards	Primary	1.83 ± 1.11	4	1.202	0.311
	Higher	3.05 ± 1.53	87		
	Vocational	2.44 ± 0.19	3		
	Secondary	3.22 ± 1.59	54		
Love of Sport	Primary	4.83 ± 2.12	4	0.642	0.589
	Higher	5.57 ± 1.26	87		
	Vocational	5.17 ± 1.26	3		
	Secondary	5.68 ± 1.34	54		

**Table 3:** Motivational results of sports volunteers from Poznan by place of residence.

Study Variables	Place of Residence	Mean ± SD	N	F	*p*
Expression of Values	large city	6.23 ± 0.95	94	1.664	0.177
	medium-sized town	6.16 ± 1.13	8		
	small town	5.96 ± 0.80	7		
	village	6.55 ± 0.64	39		
Patriotism	large city	3.40 ± 1.49	94	3.664	0.014 *
	medium-sized town	3.73 ± 1.18	8		
	small town	4.71 ± 1.74	7		
	village	4.25 ± 1.82	39		
Interpersonal Contacts	large city	5.59 ± 0.96	94	4.431	0.005 **
	medium-sized town	5.55 ± 1.62	8		
	small town	6.11 ± 0.89	7		
	village	6.21 ± 0.69	39		
Career Orientation	large city	5.27 ± 1.13	94	0.567	0.638
	medium-sized town	5.06 ± 2.18	8		
	small town	5.64 ± 1.24	7		
	village	5.49 ± 1.21	39		
Personal Growth	large city	5.38 ± 1.01	94	6.543	0.000 **
	medium-sized town	5.11 ± 1.56	8		
	small town	4.80 ± 1.23	7		
	village	6.09 ± 0.76	39		
Extrinsic Rewards	large city	2.99 ± 1.36	94	3.445	0.018 *
	medium-sized town	1.92 ± 1.14	8		
	small town	4.29 ± 2.21	7		
	village	3.27 ± 1.72	39		
Love of Sport	large city	5.41 ± 1.27	94	2.252	0.085
	medium-sized town	5.29 ± 1.60	8		
	small town	5.67 ± 1.86	7		
	village	6.03 ± 1.17	39		

**Table 4:** Motivational results of sports volunteers from Poznan by age and BMI.

Study Variables		r	*p*
Expression of Values	Age	0.134	0.124
Patriotism		0.280	0.001 **
Interpersonal Contacts		0.006	0.941
Career Orientation		−0.135	0.102
Personal Growth		0.091	0.271
Extrinsic Rewards		–0.006	0.943
Love of Sport		0.010	0.905
Expression of Values	BMI	−0.052	0.530
Patriotism		−0.076	0.358
Interpersonal Contacts		0.053	0.520
Career Orientation		−0.079	0.338
Personal Growth		0.037	0.660
Extrinsic Rewards		−0.004	0.962
Love of Sport		−0.205	0.012 *

## Data Availability

The original contributions presented in this study are included in the article. Further inquiries can be directed to the corresponding author.
